# Cross-Cancer Transfer Learning for Gastric Cancer Risk Prediction from Electronic Health Records

**DOI:** 10.3390/diagnostics15243175

**Published:** 2025-12-12

**Authors:** Daeyoung Hong, Jiung Kim, Jiyong Jung

**Affiliations:** School of Software Convergence, Myongji University, Seoul 03674, Republic of Korea; jiung8758@gmail.com (J.K.); jiyongj954@gmail.com (J.J.)

**Keywords:** gastric cancer, electronic health records, prediction model, machine learning, transfer learning

## Abstract

**Background**: Timely identification of individuals at elevated risk for gastric cancer (GC) within routine care could enable earlier endoscopy and referral. We posit that cancers within the gastrointestinal/hepatopancreatobiliary spectrum share signals that can be leveraged via transfer learning on electronic health records (EHRs) variables. **Methods**: We developed a cross-cancer transfer learning framework (TransferGC) on structured EHR data from the MIMIC-IV database, including 508 GC cases in the target cohort, that pretrains on non-gastric gastrointestinal/hepatopancreatobiliary cancers (colorectal, esophageal, liver, pancreatic) and then adapts to GC using only structured variables. We compared transfer variants against strong non-transfer baselines (logistic regression, XGBoost, architecturally matched multilayer perceptron), with area under the receiver operating characteristic curve (AUROC) and average precision (AP) as primary endpoints and F1 and sensitivity/specificity as secondary endpoints. **Results**: In the full-label setting, Transfer achieved AUROC 0.854 and AP 0.600, outperforming logistic regression (LR), extreme gradient boosting (XGB) and improving over the scratch multilayer perceptron (MLP) in AUROC (+0.024) and F1 (+0.027), while AP was essentially tied (Transfer 0.600 vs. MLP 0.603). As GC labels were reduced, Transfer maintained the strongest overall performance. **Conclusions**: Cross-cancer transfer on structured EHR data suggests a sample-efficient route to GC risk modeling under label scarcity. However, because all models were developed and evaluated using a single-center inpatient dataset, external validation on multi-center and outpatient cohorts will be essential to establish generalizability before deployment. If confirmed in future studies, the proposed framework could be integrated into EHR-based triage and clinical decision support workflows to flag patients at elevated GC risk for timely endoscopy and specialist referral.

## 1. Introduction

Gastric cancer (GC) continues to pose a major challenge to global health, ranking as the fifth most frequently diagnosed malignancy and the fourth leading cause of cancer-related death worldwide [1]. Prognosis remains strongly dependent on the stage at diagnosis, with 5-year survival rates exceeding 70% for localized disease but dropping below 10% for metastatic GC [2].

Against this background, artificial intelligence (AI) is being explored as a tool to support screening and diagnosis across a broad spectrum of surgical diseases, including benign and malignant gastrointestinal disorders. AI-based algorithms are being incorporated into screening pathways and perioperative decision-making in surgical oncology to improve detection efficiency and diagnostic accuracy [3]. These developments illustrate the potential of AI-driven predictive models to facilitate earlier detection of GC and to optimize the use of resource-intensive investigations such as upper endoscopy in high-risk populations.

Harnessing routinely collected electronic health record (EHR) data for earlier GC identification could improve outcomes by enabling timely endoscopy and treatment. Structured EHR signals—such as demographics, diagnoses, and laboratory data—represent an attractive source for data-driven cancer screening [4,5]. However, EHR-based model development can face persistent challenges, including incomplete or missing records, irregular sampling intervals, and the overall complexity of clinical documentation. Moreover, ethical and regulatory requirements—such as institutional approvals or patient consent—can further limit the volume of analyzable data, leading to relatively small, heterogeneous datasets for model training.

For gastric cancer prediction, machine learning models such as logistic regression and XGBoost, using routine structured variables (e.g., demographics, diagnoses, and common laboratory tests), have been investigated [5,6,7]. Despite these advances, most prior studies have focused on within-cancer prediction and have not examined whether cross-cancer regularities in structured EHR signals (e.g., anemia, inflammation, and metabolic axes) can be distilled and transferred to improve GC modeling under data scarcity.

Across gastrointestinal (GI) and hepatopancreatobiliary (HPB) malignancies, several clinical and laboratory signatures that are visible in structured EHRs recur across tumor types and are directly relevant for GC. Luminal GI tumors, particularly colorectal and gastric cancers, often present with chronic occult bleeding and iron-deficiency anemia (IDA); population-based studies using primary care or administrative data show that anemia and low hemoglobin are strong early signals for colorectal cancer, and clinical practice updates emphasize that otherwise unexplained IDA in adults should prompt evaluation of the upper and lower GI tract for malignancy [8,9,10,11]. In chronic liver disease and primary liver cancer, IDA frequently co-occurs with systemic inflammation and malnutrition, and biomarkers such as albumin, C-reactive protein, neutrophil–lymphocyte ratio, and platelet–lymphocyte ratio are prognostic in both liver cancer and GC [12,13,14,15]. Metabolic syndrome and dysglycemia form another shared axis: large cohort and meta-analytic studies link diabetes and metabolic syndrome not only to colorectal and pancreatic cancer risk but also to increased GC incidence [16,17,18,19,20]. Taken together, these data suggest that routinely collected features such as complete blood count indices, basic chemistries, and International Classification of Diseases (ICD) codes for IDA, liver disease, diabetes, and other metabolic comorbidities encode biologically meaningful cross-cancer information. A multi-cancer pretraining stage can therefore exploit this shared structure to learn representations that are transferable to GC.

Deep learning (DL) provides a principled way to learn non-linear interactions from tabular EHR variables and to distill noisy laboratory measurements into task-relevant representations. When labels for GC are limited, transfer learning and multi-task learning can mitigate data scarcity by first inducing shared, cancer-agnostic representations on related cancers and then adapting them to GC [21,22,23]. In this paradigm, DL primarily functions as a representation learner that captures cross-cancer laboratory signatures expected to recur in GC, thereby improving sample efficiency and stability during adaptation.

Recent work has increasingly explored self-supervised pretraining and EHR foundation models, which learn general-purpose patient representations using contrastive or masked-feature objectives before task-specific fine-tuning [24,25,26]. Our work is complementary to these efforts: instead of learning a general-purpose foundation model from very large unlabeled corpora, we study a supervised transfer setting in which labeled non-GC cancer cohorts are used to pre-train a neural encoder and then adapt it to GC. This design examines how effectively structured variables and cross-cancer label supervision can be leveraged for GC representation learning, while remaining compatible with future integration of self-supervised objectives.

We focus our initial modeling on routinely collected structured EHR variables (demographics, ICD-derived comorbidities, and routine laboratory tests) since these variables are ubiquitously available across care settings, standardized, and low-burden to obtain, which makes models straightforward to deploy in real-world workflows [4,5]. We therefore treat structured EHRs as a deployment-friendly starting point; integrating imaging and notes is an important direction for future work once the structured-only baseline is firmly established.

Accordingly, we aimed to investigate a cross-cancer transfer-learning paradigm with deep learning based on EHR data: we pre-train a shared multilayer-perceptron (MLP) backbone on non-gastric GI/HPB cancers using routinely collected EHR variables (demographics, diagnoses/ICD codes, and laboratory values) and then adapt it to GC via fine-tuning. We evaluate this hypothesis retrospectively in the MIMIC-IV v3.1 EHR database [27], restricting inputs to structured EHR data available without using diagnostic endoscopy or pathology.

From a clinical perspective, the proposed framework is especially relevant for health systems that see relatively few GC cases but routinely manage other gastrointestinal and hepatopancreatobiliary malignancies. In such settings, our study shows how existing structured EHR data and non-GC cancer cohorts can be reused to build a risk model for GC under label scarcity without requiring new data collection. Beyond GC, the same cross-cancer transfer framework can be directly adapted to other underrepresented cancers or rare tumor subtypes where labeled data are limited but related malignancies are more prevalent.

From a methodological standpoint, our framework is deliberately aligned with established multi-task and transfer-learning formulations [21,22,23]. Classical multi-task learning typically trains a single model jointly on multiple tasks and reports performance averaged across those tasks. In contrast, we treat colorectal, esophageal, liver, and pancreatic cancers as auxiliary source tasks that are used only during pretraining, and we evaluate performance exclusively on a single target task (gastric cancer) under varying degrees of label scarcity. Our primary goal is to ask an applied question: to what extent can cross-cancer supervision on routine laboratory and comorbidity profiles improve GC risk modeling under label scarcity when using only structured EHR variables? Accordingly, the contribution of this work is mainly empirical and translational: we provide the assessment of cross-cancer transfer on structured EHRs for GC, including (i) label-fraction sweeps that emulate low-resource GC settings, (ii) ablations on pretraining source-cancer composition, and (iii) comparisons between freezing versus full fine-tuning of the shared encoder.

## 2. Materials and Methods

### 2.1. Data Source and Ethics

We used the publicly available de-identified MIMIC-IV clinical database (v3.1) [27]. Access to MIMIC-IV was granted to all authors who completed the required CITI training, and we adhered to all PhysioNet credentialing and data use agreements, including the prohibition of data disclosure and misuse.

### 2.2. Cohort Construction and Features

In this study, we constructed cohorts not only for gastric cancer (GC) but also for multiple non-GC cancers (colorectal, esophageal, liver, and pancreatic). This design emphasizes that our approach leverages diverse cancer types for pre-training before fine-tuning on GC. Throughout, we use the notations to denote cancer types: GC = gastric cancer (target task), CC = colorectal cancer, EC = esophageal cancer, LC = liver cancer, and PC = pancreatic cancer.

For each cancer, we identified cases by the earliest qualifying ICD-9/10 diagnosis. We constructed features as follows. For cases, we anchored on the admission during which the cancer was diagnosed. For CBC and CMP laboratories, we summarized each test using the oldest available value observed within the 730 days prior to (and including) the admission start date for cases (and across the full available record for controls). This design was chosen to approximate a screening-like deployment scenario in which risk scores would be computed at relatively routine encounters rather than only immediately before diagnosis, and to harmonize feature construction across cancers with heterogeneous visit frequencies and order sets using a simple tabular representation. We acknowledge that this summary collapses longitudinal trajectories to a single snapshot and therefore does not capture intra-individual trends or variability; we revisit the implications of this choice in the Limitations.

We restricted the inputs to routinely collected, structured EHR variables. Feature selection was informed by prior gastric cancer–related studies [5,6,7] and guided by the principle of prioritizing routinely measured, low-burden variables with minimal missingness (e.g., complete blood count [CBC] and comprehensive metabolic panel [CMP] components). Demographic variables included age and sex. ICD-derived binary comorbidity indicators encompassed diabetes mellitus; obesity; alcohol use disorder; current or prior tobacco use; gastroesophageal reflux disease (GERD); dyspepsia; peptic ulcer disease; chronic gastritis; *Helicobacter pylori* infection; hypertension; history of myocardial infarction or angina; dyslipidemia or pure hypercholesterolemia; family history of cancer; protein–calorie malnutrition; post-hemorrhagic anemia; adverse effects of antineoplastic agents; dysphagia; receipt of antineoplastic chemotherapy; atrial fibrillation; iron-deficiency anemia; chronic kidney failure; coronary atherosclerosis or ischemic heart disease without angina; and hypo-osmolality or hyponatremia. Laboratory features consisted of CBC indices and basic chemistries, including hemoglobin, hematocrit, red blood cell count, white blood cell count, platelet count, mean corpuscular volume (MCV), mean corpuscular hemoglobin (MCH), mean corpuscular hemoglobin concentration (MCHC), red cell distribution width (RDW), leukocyte differential percentages (neutrophils, lymphocytes, monocytes, eosinophils, and basophils), sodium, potassium, chloride, bicarbonate, blood urea nitrogen, creatinine, glucose, and anion gap.

We then enforced a lab-complete inclusion criterion: patients with any missing value in the required laboratory feature set were excluded prior to modeling. For each cancer, controls were sampled at a fixed 1:3 case–control ratio from patients without a diagnosis of the cancer (ever), and the same lab-complete criterion was applied to controls. This filter excludes approximately 31%, 36%, 19%, 31% and 25% of initially eligible GC, CC, EC, LC and PC cohorts, respectively. In addition, this case–control design yields cohorts with one case per three controls, which partially mitigates the extreme class imbalance of GC in the full MIMIC-IV population while preserving a majority of controls. Identical preprocessing was applied to cases and controls; numerical features were standardized (z-scored) using statistics computed on the training split only.

### 2.3. Models

Our primary objective is to quantify how much transfer learning from non-gastric cancers improves early prediction of gastric cancer (GC). We first introduce the transfer learning framework, which pre-trains models on multiple non-GC cancers and subsequently fine-tunes them on GC. We then describe the non-transfer baselines trained solely on GC data for direct comparison. To isolate the effect of transfer, we enforce identical feature sets, preprocessing steps, and stratified data splits across all methods.

#### 2.3.1. Transfer MLP

Let C denote the set of non-gastric cancers used for pretraining; in our main configuration, C={CC,EC,LC,PC} (i.e., colorectal, esophageal, liver, and pancreatic cancers, respectively). For each c∈C, let $D_{c}={\left\{\left(x_{i}^{\left(c\right)},y_{i}^{\left(c\right)}\right)\right\}}_{i=1}^{N_{c}}$ be the corresponding case–control dataset (identical feature space across *c*), constructed with a case–control ratio of 1:3 (one case per three controls). Here y∈{0,1} indicates the disease status for cancer type *c* (y=1 for case with a confirmed diagnosis of type *c*, and y=0 for a control without that diagnosis).

Our deep learning model consists of a shared backbone encoder $f_{\phi }:R^{d_{\mathrm{in}}}\;\to \;R^{d_{h}}$ with model parameters ϕ and cancer-specific linear heads ${\left\{h_{\theta_{c}}\right\}}_{c\in C}$ acting on the latent representation $u=f_{\phi }\left(x\right)$ for input features *x*. Each head with model parameters $\theta_{c}=\left(w_{c},b_{c}\right)\in R^{d_{h}}\times R$ is an affine map parameterized as

$$h_{\theta_{c}}\left(u\right)=\langle w_{c},u\rangle +b_{c},$$

and produces a logit $z_{c}\left(x\right)=h_{\theta_{c}}\left(f_{\phi }\left(x\right)\right)$. The backbone encoder $f_{\phi }$ is a feed-forward network with linear blocks, batch normalization [28], ReLU, and dropout [29].

We first define the class-weighted binary cross-entropy (BCE) used throughout. Let $\sigma \left(t\right)=\frac{1}{1+\exp(-t)}$ denote the logistic sigmoid. Given a logit z∈R and a binary label y∈{0,1}, the per-sample loss is

(1) $$\ell_{\mathrm{BCE}}\left(z,y;\alpha \right)=-\alpha y\log\sigma \left(z\right)-\left(1-y\right)\log\left(1-\sigma (z)\right),$$

where α>0 is the positive-class weight; in our experiments we set α=3 to match the 1:3 case–control sampling design.

During pretraining, we jointly optimize the backbone $f_{\phi }$ and all cancer-specific heads $\Theta_{h}={\left\{\theta_{c}\right\}}_{c\in C}$ by minimizing the mean of the weighted BCE over cancer types:

(2) $$\frac{1}{\left|C\right|}\sum_{c\in C}\;E_{\left(x,y\right)∼D_{c}}\left[\ell_{\mathrm{BCE}}\left(h_{\theta_{c}}\left(f_{\phi }\left(x\right)\right),y;\alpha \right)\right],$$

with positive-class weight α=3. To reduce overfitting, we applied standard $\ell_{2}$-regularization (weight decay) with coefficient $\lambda_{\mathrm{pre}}$. Thus, the pretraining objective is

(3) $$L_{\mathrm{pre}}\left(\phi ,\Theta_{h}\right)=\frac{1}{\left|C\right|}\sum_{c\in C}E_{\left(x,y\right)∼D_{c}}\left[\ell_{\mathrm{BCE}}\left(h_{\theta_{c}}\left(f_{\phi }\left(x\right)\right),y;\alpha \right)\right]+\lambda_{\mathrm{pre}}\left({∥\phi ∥}_{2}^{2}+\sum_{c\in C}{∥\theta_{c}∥}_{2}^{2}\right).$$

In practice, model training does not update the parameters using the entire dataset at once, but rather processes smaller subsets of data called mini-batches. A mini-batch is simply a small group of samples (e.g., a few dozen patients) drawn from the full dataset, and the model parameters are updated based on the gradient of the loss function computed by using only that group. This strategy reduces memory requirements and stabilizes optimization compared to using a single sample (stochastic updates) or the full dataset (full-batch updates). During pretraining, at each optimization step we draw one mini-batch $B_{c}$ from each cancer type c∈C and compute their respective losses. The pretraining loss for a step is then obtained by averaging over all cancer types:

(4) L^pre(ϕ,Θh)=1|C|∑c∈C1|Bc|∑(x,y)∈BcℓBCE(hθc(fϕ(x)),y;α)+λpre(∥ϕ∥22+∑c∈C∥θc∥22).

In this way, each cancer-specific head $h_{\theta_{c}}$ is trained using its own mini-batch, while the backbone encoder $f_{\phi }$ receives gradients aggregated across cancers, encouraging it to learn shared representations.

*Expected effect.* Type-balanced, multi-task pretraining (Equations (3) and (4)) is expected to (i) induce cancer-agnostic risk representations in $f_{\phi }$, (ii) improve sample efficiency and stabilize optimization when fine-tuning on gastric cancer with limited labels, and (iii) mitigate label-imbalance effects through the weighted loss in Equation (1). These mechanisms are consistent with established benefits of multi-task learning and transfer learning [21,22,23,30].

For training the prediction model for GC, we introduce new GC linear head $h_{\theta_{\mathrm{GC}}}:R^{d_{h}}\;\to \;R$ whose model parameters $\theta_{\mathrm{GC}}$ are randomly initialized. For input features *x*, by using the pretrained backbone encoder $f_{\phi }$, the probability of GC is predicted by applying the sigmoid function σ to the logit $h_{\theta_{\mathrm{GC}}}\left(f_{\phi }\left(x\right)\right)$ (i.e., $\sigma (h_{\theta_{\mathrm{GC}}}\left(f_{\phi }\left(x\right)\right))$). Let $\phi^{⋆}$ denote the pretrained model parameters of backbone encoder. For fine-tuning, we first initialize the model parameters of backbone ϕ to $\phi^{⋆}$, and then jointly optimize the backbone $f_{\phi }$ and the GC head $h_{\theta_{\mathrm{GC}}}$ by minimizing the weighted BCE for the GC dataset:

(5) $$L_{\mathrm{ft}}\left(\phi ,\theta_{\mathrm{GC}}\right)=E_{\left(x,y\right)∼D_{\mathrm{GC}}}\;\left[\ell_{\mathrm{BCE}}\left(h_{\theta_{\mathrm{GC}}}\left(f_{\phi }\left(x\right)\right),y;\alpha \right)\right]+\lambda_{\mathrm{ft}}\left({∥\phi ∥}_{2}^{2}+{∥\theta_{\mathrm{GC}}∥}_{2}^{2}\right),$$

where we use positive-class weight α=3 and $\lambda_{\mathrm{ft}}$ is the $\ell_{2}$-regularization coefficient for fine-tuning. Details of other hyperparameters are provided in Appendix A.

#### 2.3.2. Baselines (Non-Transfer)

We benchmark three widely used non-transfer families that reflect complementary inductive biases for structured EHR data [6,31,32]. To avoid conflating modeling choices with pretraining, all baselines use the identical feature set, preprocessing, and stratified data splits as the Transfer MLP.

##### Logistic Regression (LR)

A linear probabilistic classifier that models the log-odds of GC as an affine function of the inputs, offering a strong, interpretable tabular baseline. LR captures additive effects and provides well-calibrated risk estimates under mild assumptions, making it a common comparator in clinical prediction studies.

##### eXtreme Gradient Boosting (XGB)

An ensemble of decision trees trained by gradient boosting to approximate non-linear decision boundaries and higher-order feature interactions on tabular data. This class of models is competitive on EHR tasks due to its flexibility with mixed-scale features and robustness to outliers and sparse signals.

##### MLP Trained from Scratch (MLP)

To isolate the effect of transfer learning, we also evaluate a multilayer perceptron that is architecturally identical to the Transfer MLP’s backbone and GC head (linear blocks with batch normalization, ReLU, and dropout), and uses the same training objective, mini-batch formulation, and regularization as in fine-tuning. The only difference is that the pretraining stage on non-GC cancer datasets is omitted: all parameters are randomly initialized and optimized solely on the GC dataset. Conceptually, this baseline serves as an ablation of the Transfer MLP in which the pretraining component is removed; thus, any performance gap directly quantifies the benefit of transfer.

### 2.4. Statistical Analysis

For each cancer type, we first divided the subjects into training and test sets, using an 85–15% split. We further split the training set so that 20% of it was used as a validation set. In all splits, stratified sampling was performed to ensure that the proportion of cases and controls remained consistent across the training, validation, and test sets. For a given hyperparameter setting, we trained model parameters using the training set, and computed the model performances for the validation and test sets. The evaluation metrics included the area under the receiver operating characteristic curve (AUROC), average precision (AP), sensitivity, specificity, and F1-score. To assess calibration of the GC risk models, we additionally constructed reliability diagrams on the held-out GC test set. For each model, predicted probabilities were grouped into disjoint bins on [0,1], and we plotted the observed fraction of GC cases against the mean predicted probability in each bin. We also summarized miscalibration using the expected calibration error (ECE), defined as the average absolute difference between observed and predicted risk across bins, weighted by bin size. We did not apply any additional post-hoc probability calibration procedures; all reported GC risk probabilities correspond to the raw outputs of each model trained with class-weighted binary cross-entropy. This entire procedure was repeated 10 times with different data splits. To optimize hyperparameters, we computed the mean AUROC on the validation set across these repetitions and selected hyperparameters based on this mean AUROC. The final model performance was then evaluated on the independent test split, and we report the mean performance across the 10 repetitions as the final test result. To quantify both model stability and uncertainty, we also report standard deviations or 95% confidence intervals across repetitions. In addition, we computed 95% confidence intervals (CIs) for AUROC using patient-level bootstrap resampling of the test set with 2000 resamples. To formally assess whether differences in AUROC between models sharing the same test set were statistically significant, we also applied DeLong’s test for ROC curves to obtain *p*-values for pairwise AUROC comparisons between the Transfer model and each baseline (LR, XGB, and scratch MLP).

Metrics such as AUROC and average precision (AP) were computed directly from the continuous prediction scores without applying any threshold. For threshold-dependent measures, operating points were determined using the validation split and then applied to the independent test split. Specifically, the threshold for the F1-score was chosen as the point that maximized the validation F1, and this same threshold was used to compute the F1-score on the test set. For sensitivity and specificity, following the approach in [4], we selected the threshold that provided the best balance between the two metrics—that is, the point on the validation receiver operating characteristic (ROC) curve that was closest to the ideal point (FPR = 0, TPR = 1) on the validation ROC curve. All reported sensitivity and specificity values on the test set were based on this validation-derived threshold. These validation-derived operating points were used to provide a reproducible summary of model performance in this retrospective analysis and are not intended to represent fixed clinical decision cut-offs.

To provide explanations of the Transfer model, we additionally computed SHapley Additive Explanation (SHAP) values. SHAP attributions for the neural network were obtained using the DeepLIFT-based approximation for deep models [33,34]. For each patient in the held-out GC test set, SHAP values were calculated for every input feature, and feature importance was summarized as the mean absolute SHAP value across patients. The larger values indicate a greater overall influence of that feature on the model’s predicted GC risk.

Taken together, class imbalance between cancer cases and controls was handled using a combination of (i) a nested case–control design with a fixed 1:3 case–control sampling ratio, (ii) stratified sampling to preserve the case proportion across training, validation, and test splits, (iii) class-weighted binary cross-entropy with positive-class weight α=3 during model training, and (iv) evaluation based on metrics that are less sensitive to class prevalence, including AUROC and average precision. Threshold-dependent metrics such as sensitivity, specificity, and F1-score were computed using thresholds selected on the validation set, which reduces instability due to class imbalance when evaluating on the independent test set.

To facilitate reproducibility, we will release the implementation in a public GitHub repository at https://github.com/mrailab/GCTransfer.git. All experiments were conducted using Python 3.13 and PyTorch 2.7. Because access to MIMIC-IV requires credentialing and a data-use agreement, the repository will be designed to work with any user who has approved access to MIMIC-IV.

## 3. Results

### 3.1. Cohort Overview

We constructed case–control cohorts for GC (target) and four source cancers (CC, EC, LC, PC) at a 1:3 ratio. Table 1 summarizes sample sizes and demographics. Across cohorts, the sex distribution was approximately balanced overall (weighted mean ≈50% male), and age medians clustered around the late 50s (57–60 years) with interquartile ranges spanning roughly the early 40s to the early 70s. Within the pretraining pool, EC constitutes the smallest cohort (364 cases, 1092 controls), whereas PC provides the largest case count (1467), followed by LC (1432) and CC (1296). In aggregate, the non-GC sources contribute 4559 cases and 13,677 controls (total 18,236), compared with 508 and 1524 for GC (total 2032); thus, the pretraining pool supplies approximately nine times more labeled samples than the GC task alone, a scale difference pertinent for transfer learning before adaptation to GC.

### 3.2. Comparison of Models

As summarized in Table 2, the Transfer model achieved the highest discrimination and the strongest overall operating profile on the full-label setting. Transfer obtained the top AUROC (0.854) and the highest F1 (0.636), with the best specificity (0.786). Average precision (AP) was comparable to the scratch MLP (Transfer 0.600 vs. MLP 0.603) and higher than LR (0.518) and XGB (0.549). Sensitivity for the MLP (0.782) slightly exceeded that of Transfer (0.768). Overall, relative to the scratch MLP, Transfer improved AUROC by +0.024 and F1 by +0.027 (and specificity by +0.048), at the cost of a marginally lower AP (−0.003), indicating that pretraining on related non-GC cancers yields more informative representations for GC risk despite a small AP trade-off.

Across the ten repetitions, the standard deviations of test AUROC were small for all models (≤0.01), suggesting stable performance estimates rather than isolated favorable runs. For AUROC, the 95% bootstrap confidence interval for the Transfer model (0.816–0.896) was shifted upward relative to that of the scratch MLP (0.781–0.883), consistent with a modest but reproducible gain. Because the held-out GC test set contains a limited number of positive cases, these bootstrap CIs are relatively wide and partially overlapping across models. In this sense, the width of the CIs primarily quantifies how much the performance would fluctuate if a similarly sized test cohort were resampled, while the consistent shift in the mean AUROC toward the Transfer model suggests a small but robust advantage.

Pairwise AUROC comparisons using DeLong’s test mirrored this pattern. On the common GC test set, the Transfer model achieved significantly higher AUROC than LR and XGB (p=0.0023 and p=0.0433, respectively), whereas its advantage over the scratch MLP did not reach conventional statistical significance (p=0.1231). Given the moderate size of the held-out GC test set and the limited number of positive cases, this non-significant result for the Transfer versus MLP comparison likely reflects both the modest effect size and the finite-sample uncertainty rather than the absence of any performance difference.

**Figure 1:**
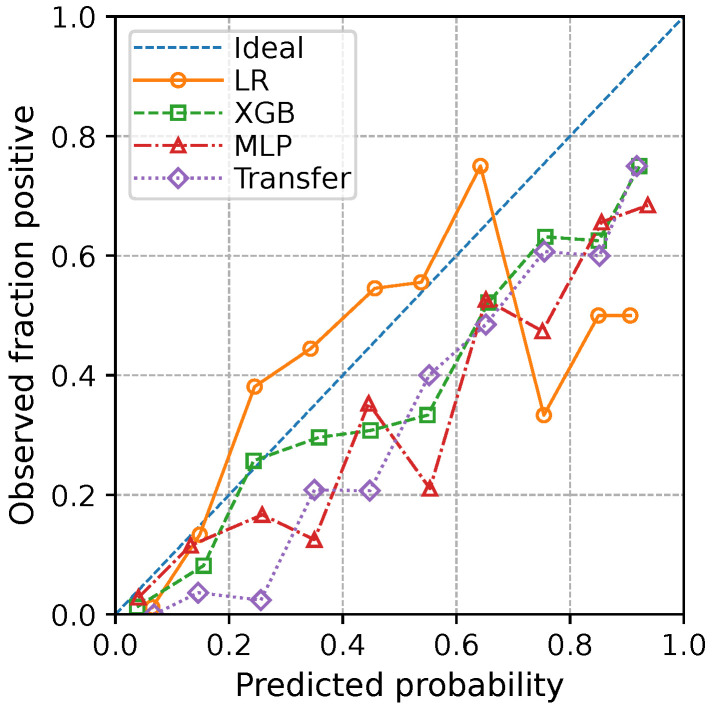
Reliability diagram showing observed GC incidence versus mean predicted probability. The diagonal line denotes perfect calibration.

With respect to calibration, the reliability diagram in Figure 1 shows that all four models are reasonably well aligned with the identity line overall. The non-linear models (XGB, MLP, and Transfer) tend to occupy slightly over-confident regions, particularly at higher predicted probabilities, whereas the logistic regression (LR) model remains more conservative. Consistently, the expected calibration error (ECE) was smallest for LR (0.072), followed by XGB (0.089) and the scratch MLP (0.119), with the Transfer model exhibiting the largest ECE (0.161). Thus, although the Transfer model achieves the best discrimination, its predicted risks are somewhat more over-confident than those of LR and may benefit from post hoc recalibration before deployment.

### 3.3. Model Performance Under Reduced GC Training Labels

**Figure 2:**
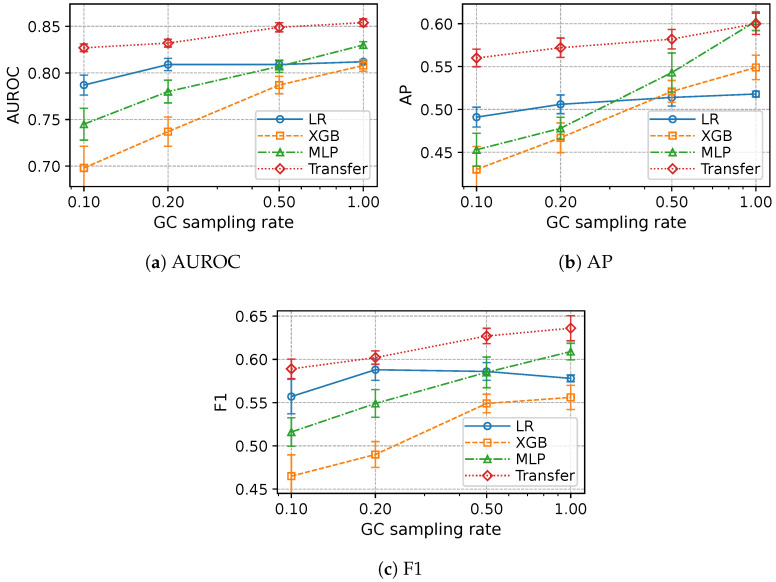
Performance across the GC sampling rate *r*. Lower *r* simulates fewer labeled GC training cases. Error bars denote 95% confidence intervals across repetitions.

As summarized in Figure 2 (AUROC, AP, and F1 panels), we varied the GC sampling rate r∈{1.0,0.5,0.2,0.1} to quantify robustness under limited GC labels. For each *r*, we applied stratified sampling at proportion *r* on the GC training partition (preserving the case:control ratio), while keeping the GC validation and test partitions fixed. Non-GC source datasets for pretraining and all preprocessing were unchanged across *r*. All models (LR, XGB, MLP-from-scratch, and Transfer) were then trained on the resulting *r*-subsampled GC training set for that rate. The Transfer model always used the same pretraining configuration on non-GC cancers and was fine-tuned on the *r*-subsampled GC training set.

As *r* decreases, all models show the expected degradation in AUROC, AP, and F1 due to reduced sample size (see Figure 2). Across all rates, the Transfer model maintains the best discrimination and precision–recall performance, with the absolute gains most pronounced at the smallest *r*. These findings indicate that pretraining on related non-GC cancers consistently improves GC risk modeling across a wide range of labeled-data regimes, with the largest improvements in AP and F1 under the scarcest-label settings. When visualized with repetition-level confidence intervals in Figure 2, this effect is also reflected in model stability. As *r* decreases, the error bars for LR, XGB, and the scratch MLP can widen, indicating increasing variability across repeated training runs. In contrast, the Transfer model maintains relatively narrow and nearly constant intervals over *r*, suggesting that cross-cancer pretraining yields more stable performance estimates under label scarcity and reduces sensitivity to sampling variability in the GC training cohort.

### 3.4. Effect of Pretraining Source Composition

To examine how pretraining source composition influences transfer performance, we repeated pretraining using one source cancer at a time and compared the results to the model pretrained on all four non-GC cancers (CC, EC, LC, PC). As shown in Table 3, single-source transfer models achieved AUROC values in the 0.828–0.841 range and F1 in the 0.594–0.632 range, with AP between 0.544 and 0.578. Among the single sources, CC yielded the highest F1 (0.632), while EC gave the highest AP (0.578). The multi–source model using CC + EC + LC + PC achieved the best overall operating profile (AUROC 0.854, F1 0.636) and an AP of 0.600. These results suggest that combining heterogeneous sources offers a modest but consistent benefit in discrimination and overall operating balance. While the multi-source model performed best in this experiment, we do not claim that this combination is universally optimal. Instead, these findings suggest that leveraging multiple heterogeneous source cancers can provide complementary EHR-visible signals, and that carefully balancing or selecting diverse sources offers potential to further improve generalization for gastric cancer prediction.

### 3.5. Freezing Versus Full Fine-Tuning Under Limited GC Labels

We investigated whether a pretrained backbone should remain fixed during adaptation to gastric cancer (GC) or be allowed to update. Although our pretraining sources (CC/EC/LC/PC) are related to GC, their feature distributions and label–feature couplings are not identical; some degree of source-to-target shift is therefore expected. In such settings, transfer learning and multi-task learning can confer advantages by first inducing shared, cancer-agnostic representations and then adapting them to the target task [21,22,23]. The practical question, however, is whether adaptation should occur only in a newly introduced GC head (with the backbone frozen; FT-Head) or across all layers (with the backbone unfrozen; FT-Full).

**Figure 3:**
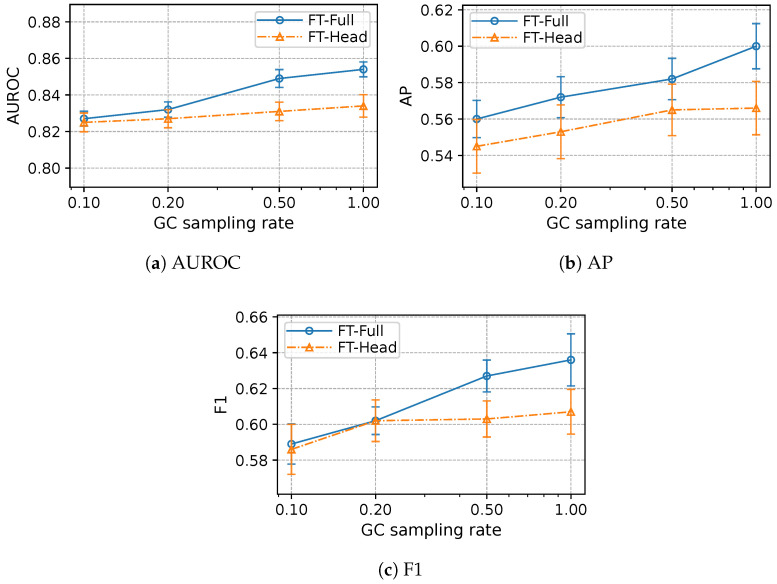
Effect of freezing vs. full fine-tuning across GC training label fractions *r*. Error bars denote 95% confidence intervals across repetitions.

To answer this, we compared FT-Head and FT-Full across GC training-label rates r∈{1.0,0.5,0.2,0.1}: FT-Head, which freezes the pretrained backbone and trains only a new linear GC head, and FT-Full, which jointly fine-tunes the backbone and the GC head. Both variants shared the same pretraining on CC + EC + LC + PC, identical features, preprocessing, and stratified splits. Figure 3 summarizes discrimination and precision–recall performance. Overall, full fine-tuning (FT-Full) outperformed freezing (FT-Head) at every label fraction, with larger gains as more GC supervision was available.

These results are consistent with two complementary mechanisms. First, allowing the backbone to update helps realign the shared representation to GC-specific covariances, improving ranking and precision–recall when the target signal is sufficiently informative [22,23]. Second, when labels are extremely scarce, freezing the backbone reduces the effective degrees of freedom, tempering variance and occasionally narrowing the gap on threshold-sensitive metrics, even if overall discrimination remains similar.

In light of the observed margins at moderate-to-higher label availability and for conceptual consistency, we use FT-Full as the primary transfer setting, using FT-Head as an ablation to quantify the value of updating the representation during adaptation.

### 3.6. Feature Importance Patterns (SHAP)

**Figure 4:**
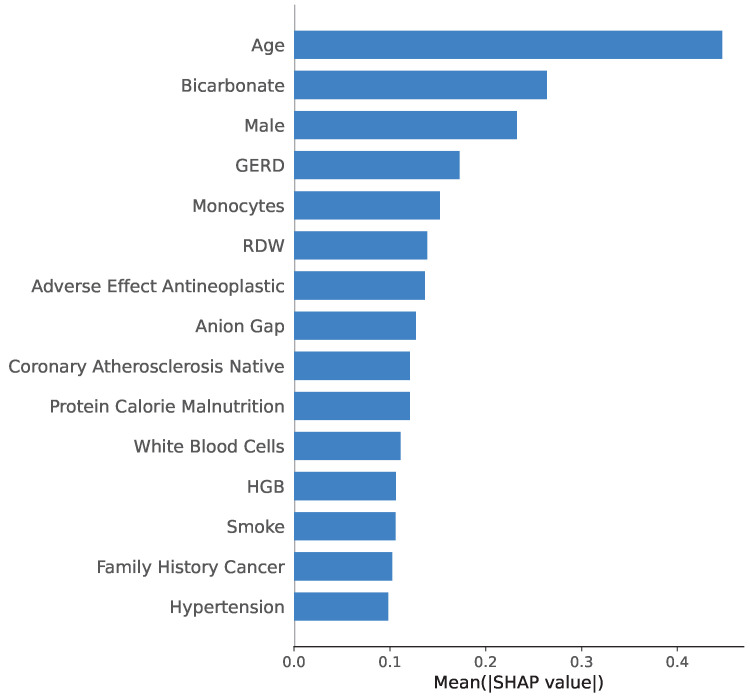
Mean absolute SHAP values (computed using a DeepLIFT-based SHAP approximation) for the Transfer model.

Global feature importance for the Transfer model is summarized in Figure 4 using mean absolute SHAP values computed on the held-out GC test set. The top 15 features by mean absolute SHAP value comprised comorbid and exposure-related variables (hypertension, smoking status, family history of cancer, gastroesophageal reflux disease, protein–calorie malnutrition, coronary atherosclerosis, and adverse effects of antineoplastic agents), blood-based indices from the complete blood count (hemoglobin, white blood cell count, red cell distribution width, and monocyte percentage), metabolic/acid–base markers (anion gap and bicarbonate), and basic demographics (age and male sex). These results indicate that the model relies on a mixture of demographic, comorbidity, and routine laboratory variables.

## 4. Discussion

In this retrospective study using de-identified structured EHR data from MIMIC-IV v3.1 [27], a transfer learning strategy that pre-trains a multilayer perceptron on non-gastric cancers (CC/EC/LC/PC) and then adapts to GC achieved the best overall performance among strong non-transfer baselines. On the full GC label regime (r=1.00), Transfer attained the highest AUROC and F1 (Table 2); AP was essentially tied with the scratch MLP (Transfer 0.600 vs. MLP 0.603). When we progressively reduced GC training labels (r↓), Transfer preserved a consistent AUROC advantage and typically higher AP—with the largest margins under the scarcest labels (Figure 2; e.g., at r=0.1, Transfer vs. scratch MLP: AUROC 0.827 vs. 0.745, AP 0.560 vs. 0.453, F1 0.589 vs. 0.516). We also observed that full fine-tuning generally outperformed frozen-backbone adaptation once modest GC supervision was available, while freezing remained competitive at the most extreme data scarcity (Figure 3). Finally, varying the composition of pretraining sources showed that combining four sources (CC + EC + LC + PC) produced the best overall performance—raising AUROC and F1 over the scratch MLP and yielding an AP essentially on par with it (Table 3).

Our findings align with prior observations that risk signals visible in structured EHR data recur across GI/HPB malignancies and therefore can act as transferable supervision for GC [4,9,10,12,13,14,18,19,20,35]. While earlier GC prediction efforts have largely optimized within-cancer models using routine variables [5,6,7], our results suggest that cross-cancer pretraining helps distill cancer-agnostic representations from noisy labs and comorbidities, which then transfer to GC with improved sample efficiency. This is consistent with established benefits of multi-task and transfer learning on related tasks [21,22,23].

Our findings are consistent with prior reports that transfer learning improves oncologic prediction under label scarcity across various data modalities. In medical imaging, models pre-trained on large source datasets and then fine-tuned on smaller tumor-specific cohorts routinely yield higher discrimination and better generalization [36]. In omics, cross-cancer transfer learning improves survival or progression prediction [37,38]. Our cross-cancer transfer on structured EHR variables parallels these patterns and suggests that cancer-agnostic signals learned from routine labs and comorbidity profiles can be reused to stabilize GC modeling when labels are limited.

We note that the absolute improvement in AUROC when comparing Transfer with the scratch MLP is modest (Table 2; AUROC 0.854 vs. 0.830). As detailed in the Results section, these gains are accompanied by improvements in F1 (0.636 vs. 0.609) and specificity (0.786 vs. 0.739), while sensitivity remains broadly similar (0.768 vs. 0.782). Despite the moderate size of the held-out GC test set, the observed differences are consistent across repeated train–test splits and bootstrap-based uncertainty estimates. Thus, we do not claim a dramatic effect size at the population level, but rather a small and reproducible shift in the operating characteristics of the model. The main value of the proposed framework lies in its stability and behavior under label scarcity. As GC training labels are reduced, the relative advantage of Transfer becomes more pronounced in AUROC, AP, and F1 (Figure 2), suggesting that pretraining on related cancers yields a more stable operating profile when data are limited. In line with this, DeLong’s test showed statistically significant AUROC improvements of the Transfer model over LR and XGB, while the comparison versus the scratch MLP did not reach conventional significance (p=0.1231), which is compatible with a small effect size and limited statistical power given the moderate GC test cohort size.

From a clinical standpoint, even such modest but consistent gains in discrimination and precision may still be relevant when predictions are used to prioritize patients for resource-intensive investigations such as upper endoscopy. The Transfer model achieves similar sensitivity but higher specificity and F1 than the scratch MLP, implying that, for a fixed endoscopy capacity, a slightly larger fraction of procedures would be directed toward patients who truly have GC and fewer toward those at very low risk. In high-volume settings where only a minority of inpatients can be referred for endoscopy, small improvements in the concentration of true GC cases within the high-risk stratum could translate into earlier diagnosis or fewer unnecessary procedures.

The operating points reported in this study (e.g., the thresholds used to summarize F1, sensitivity, and specificity) were selected on the validation set to provide a single, reproducible basis for internal model comparison. They should therefore be interpreted as illustrative examples of model behavior under specific thresholds, rather than as recommended clinical cutoffs. In practice, any deployment would require choosing a risk threshold τ in close collaboration with clinicians and service planners, taking into account local GC prevalence, endoscopy capacity, and the relative harms of false negatives and false positives. A pragmatic approach would be to first specify an acceptable lower bound on sensitivity (for example, requiring that at least 90% of GC cases over the prediction horizon are flagged as “high risk”). For each candidate threshold that meets this condition, one can then calculate simple quantities such as the expected number of endoscopies required per additional cancer detected [39]. These summaries can be compared across thresholds, taking into account local resources and the expected case mix, to select a clinically acceptable operating point. In centers with very limited endoscopy availability, a higher threshold τ might be chosen to prioritize specificity and limit the number of referrals, whereas screening programs or high-risk clinics may deliberately adopt a lower threshold to minimize missed cancers even at the cost of more false-positive procedures.

We did not perform a formal decision-curve or clinical-impact analysis in this work, and our conclusions are therefore limited to conventional discrimination and classification metrics. Future studies should quantify the net clinical benefit of using the proposed transfer-learning model to guide endoscopy referrals, for example by comparing the number of additional GC cases detected and the number of endoscopies avoided per 1000 patients across a range of clinically plausible risk thresholds.

From a calibration standpoint, our reliability and ECE analyses indicate that all four models achieve broadly acceptable calibration for GC risk estimation, with the Transfer model showing slightly more over-confident predictions than the logistic regression baseline. Such over-confidence could in principle be addressed by post hoc recalibration on an external or prospective local cohort while holding the original model parameters fixed. Concretely, one could fit a calibration model that maps the Transfer model’s risk score or its logit to a recalibrated probability, for example by updating only the intercept to match the overall GC incidence (recalibration-in-the-large) or by fitting a monotone non-parametric model such as isotonic regression [40,41]. In this setup, the Transfer model continues to provide the ranking of patients from lower to higher GC risk, and the separate calibration model aligns the predicted probabilities with the observed GC incidence in the target population.

We hypothesize three complementary mechanisms behind the observed gains: (i) *representation sharing*—pretraining encourages the backbone to encode recurring EHR patterns that GC also exhibits; (ii) *regularization under scarcity*—pretraining reduces the effective hypothesis space, aiding generalization when GC labels are limited; and (iii) *optimization stability*—a pretrained initialization improves convergence to better optima compared with training from scratch. The superiority of full fine-tuning over freezing at moderate data availability suggests that some GC-specific re-alignment of shared features is beneficial, whereas freezing can mitigate variance when labels are extremely scarce, producing similar discrimination with fewer trainable degrees of freedom. These observations match classic transfer learning trade-offs between bias and variance [22,23]. Consistent with this interpretation, the repetition-level confidence intervals in Figure 2 remain relatively tight and nearly invariant across GC sampling rates for the Transfer model, whereas most baselines exhibit visibly larger dispersion at lower rates. This pattern supports the view that pretraining acts as an implicit regularizer that dampens sensitivity to sampling noise in small GC cohorts.

Compared to single-source pretraining choices (CC, EC, LC, PC), combining all four pretraining choices delivered the strongest overall balance—improving AUROC and F1 versus scratch and yielding an AP essentially on par with it (Table 3). These results imply that (i) each source cancer contributes overlapping but not identical EHR-visible signals; and (ii) diversity across sources can add incremental value. We do not claim that our specific mix is universally optimal; rather, our results motivate future work on principled source selection and weighting schemes that account for relatedness, cohort size, and label quality.

To further contextualize the model behavior, we inspected global feature importance for the main Transfer model using mean absolute SHAP values (DeepLIFT-based) on the held-out GC test set (Figure 4). Rather than being driven by a single variable, the model primarily relies on several clinically meaningful axes: (i) anemia and nutritional status, reflected by hemoglobin, red cell distribution width, and protein–calorie malnutrition; (ii) systemic inflammation and immune activation, captured by white blood cell count and monocyte percentage; (iii) metabolic and acid–base derangements, represented by anion gap and bicarbonate; (iv) cardiometabolic and oncologic comorbidities and exposures, including hypertension, smoking, family history of cancer, coronary atherosclerosis, gastroesophageal reflux disease, and adverse effects of antineoplastic agents; and (v) baseline demographic risk (age and sex). These groupings are consistent with established clinical links between iron-deficiency anemia, malnutrition, chronic inflammation, cardiometabolic burden, familial predisposition, and gastrointestinal malignancy, supporting the view that the Transfer model is relying on clinically plausible EHR signals rather than spurious artifacts.

Taken together, these observations indicate that our study should be viewed primarily as an applied instantiation of multi-task and transfer-learning principles in a gastric cancer setting, rather than as a proposal of a fundamentally new algorithm. The backbone–head architecture, class-weighted loss, and fine-tuning strategy are intentionally simple and follow standard practice in deep learning for tabular EHR data [21,22,23]. The added value lies in the systematic evaluation of cross-cancer pretraining, label scarcity regimes, frozen versus full fine-tuning variants, and source-composition effects using only deployment-ready structured EHR variables.

From a clinical standpoint, the proposed framework is not meant to replace diagnostic endoscopy, but to support triage and prioritization in routine workflows. Because it relies only on structured EHR variables that are already collected in most hospitals, it could be implemented as a background risk score to flag patients who may benefit from earlier endoscopic evaluation or closer follow-up, particularly in systems where endoscopy capacity or specialist access is limited. Earlier identification of higher-risk patients in routine care could shorten time-to-endoscopy and facilitate detection at more treatable stages, where survival is markedly better [2]. Methodologically, the cross-cancer transfer design illustrates how related malignancies can serve as source tasks to bootstrap prediction models for cancers with few labels, a scenario that arises frequently in oncology (e.g., rare tumors, early-stage subgroups, or low-incidence settings). The transfer framework may therefore be appealing for institutions with few confirmed GC cases but access to other GI/HPB cancer cohorts; in principle, pretraining on related cancers could borrow strength and stabilize GC performance, although this remains to be confirmed in external settings.

From a deployment perspective, the proposed framework is relatively lightweight. All predictors are derived from routinely collected structured EHR data (demographics, comorbid diagnoses, medications, and basic laboratory tests) that are already available in most hospital information systems. Feature construction relies on simple summary statistics over a pre-specified look-back window and does not require free-text processing or medical imaging pipelines. The final GC risk score is produced by a shallow feed-forward neural network trained on tabular inputs, which can be evaluated efficiently on standard CPU hardware and is amenable to batch scoring of large inpatient cohorts in routine EHR jobs. Although we did not perform a formal benchmarking study of computational cost, the small model size and reliance on common laboratory panels suggest that runtime is unlikely to be the main barrier to real-world use. Instead, the more critical challenges for deployment are data governance, integration into local EHR workflows, prospective calibration monitoring, and external validation in diverse care settings. In addition, consistent with recent guidance on clinical risk prediction tools, safe deployment will require careful assessment of model calibration and subgroup fairness (e.g., across age, sex, and race/ethnicity), beyond the aggregate results reported here.

This study has several limitations.

First, all experiments were conducted using MIMIC-IV, a de-identified, single-center EHR database derived largely from inpatient encounters at a U.S. tertiary academic medical center. As a result, our models were trained and evaluated in a relatively homogeneous institutional context, with shared clinical workflows, laboratory assays, coding practices, and endoscopy referral thresholds. Patient demographics, case mix, and access to care may differ substantially in outpatient settings, community hospitals, or non-U.S. health systems, leading to distributional shifts in both predictors and outcome prevalence. Therefore, the absolute risk estimates and performance metrics reported here should not be assumed to generalize to other institutions or outpatient populations without further evaluation. Real-world deployment will require rigorous external validation and, if necessary, recalibration on independent multi-center and outpatient EHR cohorts to assess robustness under domain shift and to quantify clinical impact. In a future multicenter or international study, such validation would allow us to directly quantify how well the shared encoder and transfer-learning framework transport to hospitals with different patient populations, coding systems, and laboratory workflows, including higher-incidence regions such as East Asia. If substantial performance or calibration drift is observed, the model could be adapted in a stepwise fashion, for example by recalibrating the output layer with a modest number of local cases or by fine-tuning the encoder on pooled data from participating sites. These findings would in turn guide practical integration into hospital EHR systems by clarifying whether a single global model with site-specific decision thresholds is sufficient, or whether locally adapted models are needed to support safe deployment.

Second, cases were indexed by the first GC diagnosis code, and for each CBC/CMP laboratory we summarized the oldest available measurement within the 730-day pre-index window for cases (and across the full available record for controls). This policy was intended to reduce peri-diagnostic bias, whereby laboratory values obtained during diagnostic work-up or acute decompensation near the time of diagnosis might dominate the signal, and to emulate a more screening-like setting in which risk scores are computed at routine encounters earlier in the disease course. However, by collapsing irregular longitudinal trajectories into single-time-point snapshots, this representation cannot exploit trends, volatility, or recovery patterns that often carry additional predictive information and may attenuate the discriminative capacity of the model compared with fully longitudinal approaches. Moreover, because at least one measurement of each laboratory is required, this strategy likely enriches the cohort for patients with more complete laboratory histories and higher health-care utilization, which may bias performance estimates and limit generalizability to populations with sparser testing.

Third, we restricted all analyses to a “lab-complete” cohort, requiring non-missing values for all predefined laboratory predictors within the look-back window. This design choice simplifies model development and avoids introducing additional uncertainty from ad hoc imputation; however, it also induces selection bias. In routine care, comprehensive laboratory panels are more likely to be ordered for patients with greater healthcare utilization and higher comorbidity burden, whereas individuals with fewer encounters or milder symptoms are less likely to have all tests measured. As a result, the case mix, event rate, and distribution of predictors in our training data may differ from those in a broader outpatient or low-utilization population. In our setting, this selection mechanism is likely to introduce an optimistic bias: the lab-complete cohort is enriched for patients with denser testing and more informative predictors, so the discrimination metrics (e.g., AUROC) and positive predictive value reported here may overestimate the performance that would be observed in real-world populations where laboratory testing is sparser and missingness is more prevalent. In such settings, both AUROC and, in particular, precision/recall-based measures may be lower, and model calibration may deteriorate if the model is applied without recalibration. Future work should relax the lab-complete requirement by incorporating principled strategies for handling missing data (e.g., multiple imputation or model-based imputation), so that patients with partial laboratory profiles can also contribute to model development.

In addition, because the held-out GC test set is of moderate size with a limited number of positive cases, the bootstrap confidence intervals are relatively wide and partially overlapping across models, which reflects finite-sample uncertainty. Larger external cohorts will be required to narrow these intervals and to more precisely quantify the performance differences between models.

Finally, we did not evaluate subgroup fairness (e.g., performance stratified by age, sex, or race/ethnicity), and our calibration assessment was restricted to aggregate reliability diagrams and ECE on the GC test set. For a clinical risk prediction tool, however, well-calibrated risk estimates and equitable performance across key patient subgroups are essential for safe deployment; accordingly, detailed subgroup- and setting-specific calibration and fairness analyses, ideally using external multi-center cohorts, will be required before any clinical use.

Several extensions appear promising. (i) Move beyond static features to longitudinal encoders that incorporate trends, rates of change, and testing frequency. (ii) Combine supervised multi-task pretraining with self-supervised objectives on large unlabeled EHR (e.g., masked feature modeling) to further improve sample efficiency. (iii) Explore domain adaptation and source reweighting to mitigate negative transfer when source–target mismatch is large. (iv) Expand external validation across health systems.

## 5. Conclusions

By pretraining a shared encoder on related GI/HPB cancers and adapting it to gastric cancer (GC) using only routine and structured EHR variables, we demonstrated that cross-cancer transfer learning can stabilize GC risk modeling under label scarcity within this single-center inpatient cohort. Rather than providing a fully validated, ready-to-deploy tool, our study should be viewed as a proof-of-concept for a potentially reusable framework whose real-world utility and generalizability will need to be confirmed in future multi-center external validation studies. Because it operates on data already collected in routine care, the approach can be embedded into EHR workflows to prioritize timely endoscopy referrals, and target follow-up in resource-constrained settings—aligning with the broader role of AI as clinician decision support in acute care. Before any clinical use, external multi-site validation and prevalence-aware calibration remain essential; nonetheless, the framework offers a practical blueprint for repurposing heterogeneous cancer cohorts to bootstrap GC models and, more broadly, a reusable foundation for early-risk identification in other underrepresented cancers.

## Figures and Tables

**Table 1:** Cohort summary for the target (gastric cancer; GC) and source cancers (colorectal, esophageal, liver, pancreatic). Sex is male proportion (%), and age is reported as median [interquartile range (IQR)].

Cohort	Cases	Controls	Male (%)	Age (Median [IQR])
GC	508	1524	51.6	58.0 [41.0–71.0]
CC	1296	3888	47.1	57.0 [39.0–71.2]
EC	364	1092	52.9	58.0 [40.0–71.0]
LC	1432	4296	53.1	58.0 [40.0–70.0]
PC	1467	4401	48.2	60.0 [42.0–72.0]

**Table 2:** Performance of models. Reported values include the mean test performance across repeated runs with standard deviation (±), and the 95% bootstrap confidence interval (parentheses).

Model	AUROC	AP	Sensitivity	Specificity	F1
LR	0.812±0.002 (0.759–0.862)	0.518	0.707	0.755	0.578
XGB	0.808±0.010 (0.760–0.851)	0.549	0.692	0.737	0.556
MLP	0.830±0.006 (0.780–0.874)	0.603	0.782	0.739	0.609
Transfer	0.854±0.007 (0.811–0.893)	0.600	0.768	0.786	0.636

**Table 3:** The performance of Transfer grouped by pre-train cancer types. Reported values include the mean test performance across repeated runs with standard deviation (±).

Pretrain Types	AUROC	AP	Sensitivity	Specificity	F1
CC	0.841±0.006	0.568	0.792	0.763	0.632
PC	0.838±0.005	0.572	0.758	0.743	0.599
LC	0.835±0.007	0.544	0.767	0.745	0.605
EC	0.828±0.005	0.578	0.705	0.779	0.594
CC + EC + LC + PC	0.854±0.007	0.600	0.768	0.786	0.636

## Data Availability

The datasets in this study are available through the MIMIC-IV database at https://physionet.org/content/mimiciv/3.1/ (accessed on 1 October 2025) with the permission of PhysioNet.

## Abbreviations

The following abbreviations are used in this manuscript:

AP	Average precision
AUROC	Area under the receiver operating characteristic curve
TPR	True positive rate
FPR	False positive rate
EHR	Electronic health record
CBC	Complete blood count
CMP	Comprehensive metabolic panel
GI	Gastrointestinal
HPB	Hepatopancreatobiliary
GC	Gastric cancer
CC	Colorectal cancer
EC	Esophageal cancer
LC	Liver cancer
PC	Pancreatic cancer
LR	Logistic regression
MLP	Multilayer perceptron
XGB	Extreme Gradient Boosting

## Appendix A. Hyperparameter Details

We optimized hyperparameters using a one-factor-at-a-time (OFAT) sweep around a central configuration, selecting settings by mean validation AUROC over 10 repetitions. Neural models used Adam [42]; mini-batch size was $\left\lfloor 0.05\cdot N_{c}\right\rfloor$ capped to [8,64] where $N_{c}$ is the data size for cancer type c∈{CC,EC,LC,PC,GC}. Complete ranges and the central configuration are provided in Table A1. The meaning of some notations used in Table A1 is as follows:

- *Architecture.* A notation such as [128,64] in Table A1 denotes the MLP backbone architecture with two hidden layers of 128 and 64 units, respectively. Each hidden block is implemented as Affine-transformation→Batch-normalization→ReLU→Dropout.
- *Dropout.* 0.2 of Dropout value in Table A1 means that, during training, each hidden unit’s activation is independently set to zero with probability 0.2 (keep-probability 0.8).

**Table A1:** Hyperparameter ranges.

Hyperparameter	Values
Architecture (layers)	[128,64],[64,32],[32,16]
Dropout	0.2 (fixed)
Learning rate in pretraining	$10^{-2},\;10^{-3},\;10^{-4}$
Pretraining weight decay	$10^{-2},\;10^{-3},\;10^{-4}$
Pretraining steps	1000,2000,5000
Learning rate in fine-tuning	$10^{-3},\;10^{-4},\;10^{-5}$
Fine-tuning weight decay	$10^{-3},\;10^{-4},\;10^{-5}$
Fine-tuning epochs	20,50,100
